# Mr. Edgar Speyer's Prize

**Published:** 1906-01-13

**Authors:** E. W. Morris


### EDITOR'S LETTER-BOX.

[Our Correspondents are reminded that prolixity is a great bar to publication, and that brevity of style and conciseness of statement greatly facilitate early insertion.]

### MR. EDGAR SPEYER'S PRIZE.

Sir,—In your article on "Mr. Edgar Speyer's Prize" in your issue of January 6, the writer does not appear to have clearly understood the conditions of the competition. Each essay was to be sent in with a motto and accompanied by a sealed envelope bearing on the outside the same motto. The envelope contained the name and address of the writer of the essay.

The King's Fund promised that the envelope of the successful competitor only should be opened; all others would be destroyed unopened, together with the essays. Nothing could be fairer. The prize was won in fair field by Mr. Hamilton.

There was a second essay which the judges thought worthy of publication, although distinctly inferior to Mr. Hamilton's. Your article insinuates that there was some difficulty in finding the writer. There was no difficulty. But for their promise, the King's Fund could have opened the envelope bearing the motto "Dum spiro, spero" and have at once discovered the identity of the writer. This, of course, they could not do. Consequently a notice was inserted in the papers. On seeing the notice I communicated with Mr. Edgar Speyer at once to say that the essay was quite at his service for publication if he thought it of sufficient interest.

I should like to say that my Board, in giving me permission to compete, gave me a perfectly free hand to say exactly what I liked; not one of the Board saw the paper. I very warmly dissent from the suggestion that more candid expressions of opinion would have been obtained had they been anonymous. It is an affront to both Hospital Committees and Hospital Secretaries.

Hospital Boards are not bound to follow the suggestions of their Secretary, but a Secretary worthy of his position would certainly be encouraged to make suggestions.

I very heartily join with you in your congratulations to Mr. Hamilton,

I am, Sir, yours truly,

E. W. MORRIS.

